# Salivary cortisol in long COVID: a marker of broader stress system and circadian rhythm dysregulation

**DOI:** 10.3389/fcimb.2025.1690698

**Published:** 2026-01-06

**Authors:** Marta Camici, Marta Franco, Lorenzo Talamanca, Manuela Petino, Jessica Paulicelli, Liliana Scarnecchia, Lucia Ciavarella, Tiziana Orzilli, Francesca Balducelli, Valentina Mazzotta, Ilaria Mastrorosa, Eleonora Cimini, Eleonora Tartaglia, Stefania Notari, Fabrizio Maggi, Enrico Girardi, Roberto Baldelli, Paolo Zuppi, Andrea Antinori

**Affiliations:** 1 Clinical and Research Infectious Diseases Department, National Institute for Infectious Diseases Lazzaro Spallanzani Istituto di Ricovero e Cura a Carattere Scientifico (IRCCS), Rome, Italy; 2 Endocrinology Clinical Unit, San Camillo Forlanini Hospital, Rome, Italy; 3 Department of Biology, Institute of Molecular Systems Biology, Eidgenössische Technische Hochschule Zürich (ETH Zürich), Zurich, Switzerland; 4 Azienda Sanitaria Locale ASL Roma 1, Rome, Italy; 5 Department of Clinical Pathology, San Camillo Forlanini Hospital, Rome, Italy; 6 Laboratory of Cellular Immunology and Pharmacology, National Institute for Infectious Diseases Lazzaro Spallanzani IRCCS, Rome, Italy; 7 Laboratory of Virology, National Institute for Infectious Diseases Lazzaro Spallanzani IRCCS, Rome, Italy; 8 Scientific Direction, National Institute for Infectious Diseases Lazzaro Spallanzani IRCCS, Rome, Italy; 9 Ordine di Malta Italia, Endocrinology Project, San Giovanni Battista Hospital, Rome, Italy

**Keywords:** circadian rhythm dysregulation, fatigue assessment scale, hypothalamic-pituitary-adrenal axis impairment, long-COVID, long-COVID hallmarks, salivary cortisol profile, biomarkers

## Abstract

**Introduction:**

Long COVID (LC) has been associated with hypothalamic–pituitary–adrenal (HPA) axis dysfunction, although findings from blood cortisol measurements remain inconsistent. We hypothesized that LC patients exhibit a disrupted diurnal cortisol rhythm and that salivary cortisol (SC) profiling may provide a more accurate assessment of HPA activity.

**Methods:**

This prospective, single-center, case–control study was conducted at a Long COVID clinic in Rome between February 2023 and March 2024 and included 96 participants evaluated at least 28 days after confirmed SARS-CoV-2 infection. LC was defined as one or more new or persistent symptoms and classified as severe when four or more of the following were present: fatigue, cognitive impairment, exercise intolerance, dyspnea, arthralgia, or dysautonomia. SC was measured at 8:00 AM, 3:00 PM, and 11:00 PM.

**Results:**

The cohort (mean age 58.1 ± 14.8 years; 60% female; all White) included 83 LC patients (80% moderate, 20% severe) and 13 asymptomatic post-COVID (APC) individuals. Compared with healthy controls, both LC and APC groups showed reduced morning SC (p<0.01), flattened diurnal variation, and elevated evening SC, indicating loss of the normal morning peak and nocturnal decline. Blood cortisol levels did not differ among groups, but LC patients had higher ACTH than APC (26 pg/mL vs 13 pg/mL; p<0.01), suggesting compensatory HPA activation. One LC patient (1.2%) was diagnosed with adrenal insufficiency.

**Discussion:**

These exploratory findings suggest a disrupted circadian cortisol rhythm in individuals after COVID-19, with altered HPA axis dynamics that may be associated with disease severity.

## Introduction

Long-COVID is a multisystem disorder characterized by symptoms such as fatigue, cognitive dysfunction, sleep disturbances, and dysautonomia that persist or appear beyond the acute phase of infection (Davis et al., 2023), and impairs daily functioning and quality of life (Mastrorosa et al., 2023). Meta-analyses estimate a global prevalence of approximatively 35%, with higher rates in South America and Europe and greater risk among unvaccinated individuals, women, and those with comorbidities (Hou et al., 2025). LC symptoms can persist for years and worsen with reinfection (Babalola et al., 2025), posing a potential long-term public health burden by accelerating aging (Lord et al., 2024). These manifestations suggest possible neuroendocrine and autonomic dysregulation, including alterations of the hypothalamic–pituitary–adrenal (HPA) axis (Camici et al., 2024), which plays a central role in stress response and immune regulation. Moreover, the symptoms of LC partially overlap with those of Myalgic encephalomyelitis/Chronic Fatigue Syndrome (ME/CFS), in which reduced morning cortisol levels have been documented (Herane-Vives et al., 2020). Several studies have investigated cortisol secretion in individuals with LC. However, findings from blood-based assessments have been inconsistent. Some reports describe hypocortisolemia and blunted adrenal responsiveness (Klein et al., 2023), whereas others find normal morning serum cortisol levels (Fleischer et al., 2024). These discrepancies likely reflect methodological differences, including small sample sizes, cross-sectional designs, and the inherent limitations of single time-point blood measurements that fail to capture circadian hormonal variability. A recent study demonstrated that chronic fatigue and sleep disturbances in LC are closely linked to disruptions of circadian rhythms, mediated by distinct molecular and cellular mechanisms triggered by SARS-CoV-2 infection (Livieratos et al., 2025). These mechanisms include dysregulation of core clock genes, mitochondrial dysfunction, and cytokine-driven neuroinflammation. Most circadian, mood-related, immune, and mitochondrial genes are transcriptionally regulated by the glucocorticoid receptor and its ligand cortisol, highlighting a potential interface between HPA axis activity and circadian homeostasis (Du et al., 2009; Livieratos et al., 2025). Cortisol follows a well-defined circadian rhythm, peaking in the early morning and declining toward the evening. Salivary cortisol (SC) profiling provides a non-invasive and dynamic measure of this rhythm and may more accurately reflect HPA axis function than plasma assays (Egawa et al., 2018).

To date, no peer-reviewed studies have comprehensively characterized circadian SC profiles in individuals with LC, and their relationship to disease severity and symptom persistence remains largely unexplored. This study aimed to characterize the circadian SC profile in individuals with LC and to compare it with that of asymptomatic post-COVID and healthy controls. We hypothesized that LC patients would exhibit a disrupted cortisol rhythm, characterized by reduced morning peaks and elevated evening levels, indicative of altered HPA axis regulation and potential adrenal impairment. Given the preliminary nature of this investigation and the limited control group (n=7), findings should be considered hypothesis-generating.

## Materials and methods

### Study design and participants

**Figure 1:**
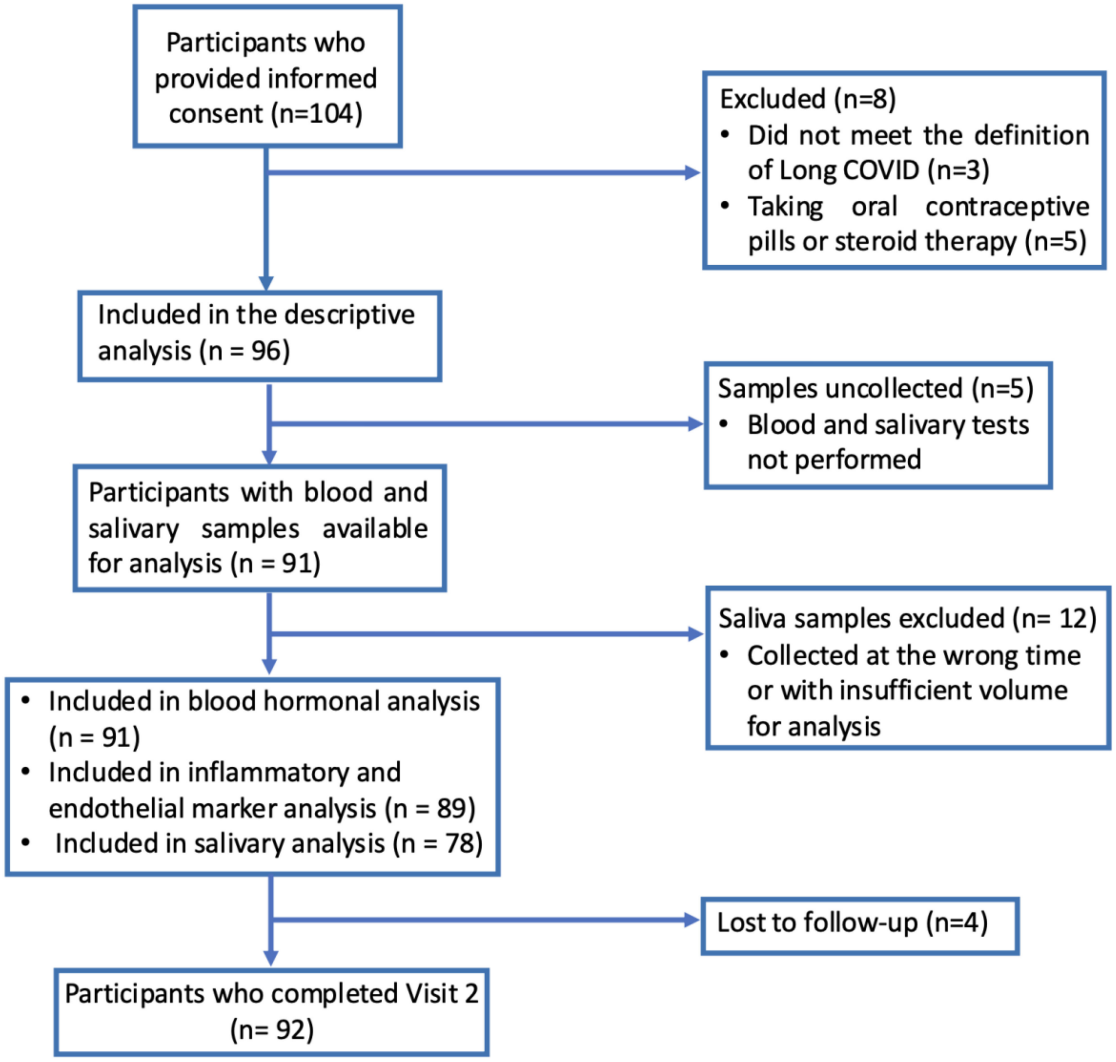
STROBE flow diagram of the study participants (STROBE, Strengthening the Reporting of Observational Studies in Epidemiology).

This prospective, single-center study was conducted at the National Institute for Infectious Diseases L. Spallanzani in Rome, Italy. The post-COVID outpatient clinic, established in 2020, initially followed all hospitalized COVID-19 patients. Over time, asymptomatic cases declined, while referrals for LC increased. Between February 2023 and March 2024, 104 consecutive patients were enrolled, referred from home, hospital wards, or the early-treatment SARS-CoV-2 clinic. Inclusion criteria were age >18, confirmed SARS-CoV-2 infection (positive swab ≥28 days before baseline), and signed informed consent. Exclusion criteria were ongoing use of systemic, injectable, or inhaled steroids; oral contraceptives; and a history of uncontrolled rheumatologic or uncontrolled endocrine disease. Uncontrolled rheumatologic disease was defined as active or unstable autoimmune conditions requiring high-dose immunosuppressive therapy or systemic steroids and associated with ongoing systemic inflammation. Uncontrolled endocrinological disorders were defined as a history of an endocrinological condition with abnormal laboratory findings. Eight participants were excluded because did not match with long-COVID definition (N = 3) or were under steroids or oral conceptive pills regimen (N = 5). Data from 96 participants were included in the descriptive analysis. Blood samples were unavailable for five participants; therefore, samples from 91 participants were analyzed (12 APC and 79 LC). For the SC analysis, patients who collected saliva at the wrong time or provided an insufficient sample for analysis were excluded. Details are shown in the study flow-chart (**Figure 1**).

### Definitions

Acute SARS-CoV-2 infection onset was defined by the first positive molecular or antigenic nasopharyngeal swab. LC was defined as ≥1 new or persistent symptom > 4 weeks post-infection. LC severity was based on the number of symptoms of interest (SOI): fatigue, cognitive deficits, poor exercise tolerance, dyspnea, arthralgia, and dysautonomia. Moderate LC was defined as 1–4 SOI, and severe LC as ≥5. Fatigue was assessed using the 10-item Fatigue Assessment Scale (FAS), which scores both physical and mental fatigue. A total score ≥22 indicates fatigue, and ≥35 indicates severe fatigue.

### Outcomes

The primary outcome was to assess daily SC variation in post–SARS-CoV-2 individuals, both symptomatic and asymptomatic, compared to healthy controls (HC). Secondary outcomes included evaluating HPA and HPT axis function, sex hormone levels, and inflammatory and coagulative markers across LC, asymptomatic post-COVID (APC), and HC groups. The study also aimed to identify risk factors associated with Long COVID development.

### Study procedures

Participants were jointly assessed by an infectious disease specialist and an endocrinologist at baseline (BL) and at a three-month follow-up (V2). At BL, detailed medical histories were recorded, including acute COVID-19 phase and diagnostic data. Clinical evaluations focused on LC symptom burden, daily functioning, and fatigue, using the Fatigue Assessment Scale (FAS).

Participants were instructed verbally and provided with a written sheet detailing the procedure for saliva collection at fixed clock times (8:00 AM, 3:00 PM, and 11:00 PM) on a typical weekday. They were advised to avoid alcohol, caffeine, physical exercise, stressful activities, and toothbrushing before sampling. Each participant recorded the exact time of collection on the vial label, and samples collected more than ±30 minutes from the scheduled time were excluded from analysis. Samples were refrigerated immediately after collection and returned during the follow-up visit. Adherence was verified through participant logs and verbal confirmation. Wake time and sleep duration were not standardized or recorded, while smoking habits were documented.

Additional diagnostic tests and specialist referrals were provided as clinically indicated. To avoid interference, after saliva and blood collection, all participants with LC were advised to follow a standardized supportive regimen comprising liposomal glutathione, vitamin C, L-arginine, a lactose- and gluten-free low-glycemic diet, yoga nidra, and individualized physical activity tailored to symptom severity. Two weeks post-BL, early morning fasting blood samples (7:30–8:30 AM) were collected for hormonal, inflammatory, and coagulative markers, including cortisol, adrenocorticotropic hormone (ACTH), dehydroepiandrosterone sulfate (DHEAS), sodium, potassium, testosterone (males), thyroid-stimulating hormone (TSH), free triiodothyronine (fT3), free thyroxine (fT4), interleukin-1β (IL-1β), IL-6, tumor necrosis factor-alpha (TNF-α), NOD-like receptor family pyrin domain-containing 3 (NLRP3), IL-8, vitamin D, C-reactive protein (CRP), D-dimer, intercellular adhesion molecule-1 (ICAM-1), and vascular cell adhesion molecule-1 (VCAM-1). A 1 µg ACTH stimulation test was performed in patients with basal cortisol 3–15 µg/dL; hypocortisolism was defined as basal <3 µg/dL or peak <18 µg/dL. SC samples were collected the day before blood sampling, refrigerated, and delivered on the test day. Saliva from seven healthy, asymptomatic controls (either COVID-negative or >6 months post-infection) was also analyzed for comparison. Individuals >6 months after SARS-CoV-2 infection were included only if they had fully recovered and reported no residual or persistent symptoms compatible with Long COVID. The >6-month cutoff was selected to minimize potential short-term post-viral HPA axis alterations. Three HC participants had never been infected with SARS-CoV-2, while the median time since previous infection among the four HC with prior COVID-19 was 627 days (IQR 127). Inflammatory and coagulative markers were analyzed in a subset of participants due to limited reagent availability. Samples were processed in the order of arrival at the laboratory, and laboratory staff were blinded to participant group. At V2, participants underwent repeat clinical and fatigue assessments to track LC symptom progression. Laboratory personnel performing hormonal and cytokine analyses were blinded to participants’ clinical classification (LC, APC, or HC) to minimize bias.

### Laboratory methods

Hormonal assays were performed on serum samples using the Atellica IM chemiluminescent immunoassay system (SIEMENS) for cortisol [reference values (rv) 4.3-22.4 µg/dL], testosterone (rv 2.5-8.5ng/ml), fT3 (rv 2.3-4.2 pg/ml), fT4 (rv 0.8-1.6 ng/dl), and TSH (rv 0.4-3.5 µU/ml). DHEAS was measured via chemiluminescence (MAGLUMI 2000 Plus, SNIBE) (rv male 18-30yrs 24-690 µg/dL; 31-50yrs 106-995 µg/dL; 51-70yrs 24-313 µg/dL; >70yrs 5-253 µg/dL)(rv female 18-30yrs 18-391 µg/dL; 31-50yrs 19-266 µg/dL; 51-70yrs 8-188 µg/dL; >70yrs 7-177 µg/dL), and ACTH (rv 7.2-63.3 pg/ml) via electrochemiluminescence (Elecsys ACTH, COBAS E411, ROCHE). Vitamin D concentrations were measured using an electrochemiluminescent immunoassay (ECLIA, Roche) (rv deficient < 25 nmol/L; insufficient 25–75 nmol/L; sufficient 75–250 nmol/L; potential toxicity > 250 nmol/L). All samples were processed within six hours. SC was collected at 8:00 AM, 3:00 PM, and 11:00 PM using Salivette devices (SARSTEDT) and analyzed with the Elecsys Cortisol II assay (ROCHE) on the COBAS E411. Inflammatory and coagulation markers were assessed in 89 participants (77 LC, 12 APC, and 18 controls). Plasma samples were obtained after centrifugation of whole blood for 10 minutes at 1,800 rpm and immediately stored at −80 °C until analysis. Plasma levels of D-dimer, NLRP3, E-selectin (E-Sel), ICAM-1, VCAM-1, IL-1β, IL-6, IL-8, and TNF-α were quantified using an automated ELISA system (ELLA microfluidic analyzer, ProteinSimple, Bio-Techne). The detection limit of these assays was 0.16 pg/mL for IL-1β, 0.28 pg/mL for IL-6, 0.19 pg/mL for IL-8, 0.30 pg/mL for TNF-α and <500 ng/ml for D-Dimer. No established clinical cut-offs exist for NLRP3 (pg/ml), ICAM-1 (pg/ml), VCAM-1 (pg/ml), or E-selectin (pg/ml). Values were therefore interpreted relative to the distribution observed in the healthy control group.

### Statistical analyses

**Figure 2:**
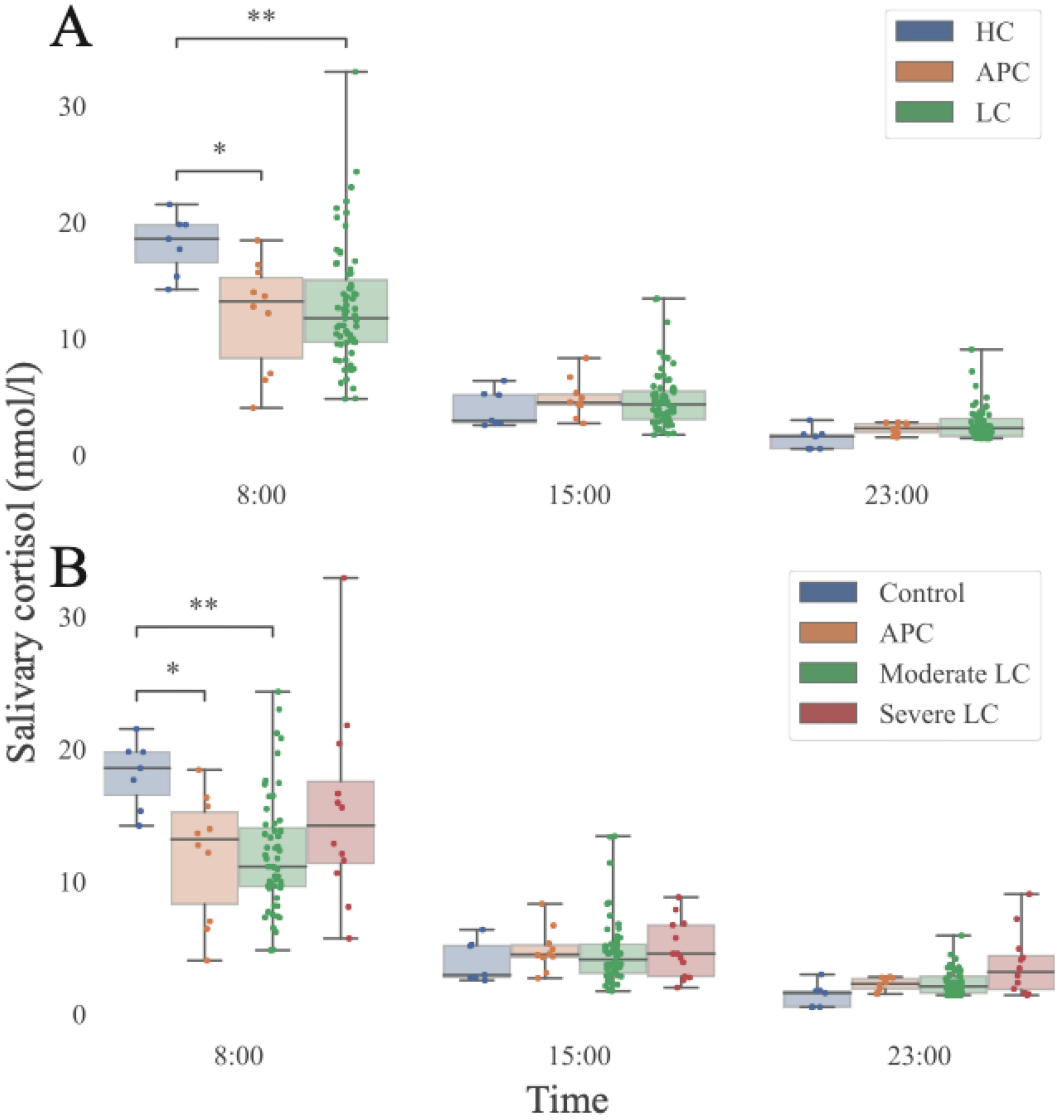
Salivary cortisol time of day dependent distribution as a function of Long COVID diagnosis and severity. **(A)** Stratification in three categories: HC, healthy control, APC, Asymptomatic Post-COVID, LC, Long-COVID. Salivary cortisol: nmol/L. **(B)** Further stratification including LC severity. HC: healthy control: asymptomatic subjects never infected by SARS-CoV-2 or presenting SARS-CoV-2 infection earlier than 6 months. Moderate: < 5 LC symptoms. Severe:≥ 5 LC symptoms. Salivary cortisol: nmol/L. We also validated these findings using a linear mixed model considering time, group (HC, APC, and either LC or Moderate LC, and Severe LC) and a time * group interaction, reported in **Supplementary Figure S1**. HC, healthy control, APC, Asymptomatic Post-COVID, LC, Long-COVID. Statistical significance was assessed using the Mann-Whitney U test and values were corrected for FDR in a measure-specific manner (*: p<0.05; **: p<0.01; HC N = 7, APC N = 10, LC N = 68, Moderate LC N = 56, Severe LC N = 12; 5^th^, 25^th^, 50^th^, 75^th^, and 95^th^ quantiles marked). Extended data in **Supplementary Table S4**.

Data from 104 patients was anonymized using patient id at collection. 96 participants were included in the analysis. We summarized the data in descriptive tables (**Table 1**) stratifying the samples in subgroups and evaluating the statistical difference across all measurements and exams. Statistical significance was assessed using non-parametric tests to avoid requiring normally distributed data. The Mann–Whitney U test was applied for all unpaired data comparisons, and the Wilcoxon signed-rank test was used to compared paired samples (for example for the visits comparison). Categorical variables were compared using Fisher’s exact test. All statistical tests were two-tailed. Variables with missing data were excluded from the analysis. Benjamini-Hochberg false discovery rate (FDR) correction was applied to each measurement across all possible pairs (HC, APC, LC, Moderate LC, and Severe LC as appropriate) to account for multiple tests but not to be overly conservative in significance estimation. For example, for the salivary cortisol time course this means that at each time point we corrected the statistical significance across the possible pairs (HC-APC, HC-LC, and APC-LC for **Figure 2a**). FDR-adjusted *p*-values < 0.05 were considered statistically significant. We tested several clinical variables (i.e., physical activity, age, gender, smoking status, body mass index, presence of more than three comorbidities, number of comorbidities, number of vaccine doses, hospitalization during the acute SARS-CoV-2 infection, home management, treatment with monoclonal antibodies or antivirals during the acute phase, and type of acute infection symptoms), to evaluate their association with the development of long COVID; the significance of these variable was not corrected for multiple tests. Statistics represented in the plots are as follows: n.s. p≥0.05, *: p<0.05, **: p<0.01, ***: p<0.001, ****: p<0.0001. All analyses were performed in Python, and statistical analysis was carried out using the Statannotations package (Vinas et al., 2023). To ensure the statistical validity of our circadian claims we performed a linear mixed model approach with the statsmodels python package (Skipper Seabold, 2010). In this linear mixed model, we considered the cortisol levels to be:

**Table 1:** Demographic and clinical characteristics.

Variables	All	APC	LC	P-value
Participants*	96	13 (14%)	83 (86%)	1
Female sex*	58 (60%)	8 (61%)	50 (60%)	0.35
Age**	58.1 (14.8)	58.5 (15.2)	58 (14.7)	0.91
BMI**	24.9 (4.6)	24.5 (5.1)	25 (4.5)	0.74
History of competitive Sports*§	47 (49%)	4 (30.1%)	43 (51.8%)	**0.04**
Smoking*§	11 (11%)	4 (30.1%)	7 (8.4%)	**0.006**
Comorbidities > 3*	59 (61%)	5 (38%)	54 (65%)	**0.02**
N&° of vaccine doses**	2.9 (1)	3.1 (1.2)	2.8 (1)	0.52
Previous SARS-CoV-2*	40 (41%)	3 (23%)	37 (44.6%)	**0.03**
FAS**	27.4 (9.7)	15.4 (6.6)	29.3 (8.7)	**<0.001**
Mental FAS**	12.7 (5.4)	7.5 (4.4)	13.5 (5)	**<0.001**
Physical FAS**	14.7 (5.1)	8 (2.7)	15.7 (4.5)	**<0.001**
Acute SARS-CoV-2 infection management				
Hospital admission	11 (11.5%)	3 (23%)	8 (9.6%)	**0.039**
Home	60 (62.5%)	7 (54%)	53 (64%)	0.15
Day hospital	24 (25%)	3 (23%)	21 (25%)	0.31
Treatment				
Oral antivirals	14 (14.6%)	4 (30%)	10 (12%)	**0.02**
Remdesivir	10 (10.4%)	2 (15.4%)	8 (9.6%)	0.13
LMWH	7 (7.3%)	2 (15.4%)	5 (6%)	**0.049**
Oxygen supplementation	9 (9.4%)	3 (23%)	6 (7%)	**0.017**
Monoclonal Antibodies	12 (12.5%)	2 (15%)	10 (12%)	0.2
IV high dose steroids	8 (10.4%)	2 (15.4%)	6 (7.2%)	0.07
NSAIDs	66 (68%)	6 (46%)	60 (72%)	**0.015**

*n (%); **Mean (SD); APC, Asymptomatic Post-COVID; LC, Long-COVID; BMI, Body Mass Index; FAS, Fatigue Assessment Scale. LMWH, Low Molecular Weight Heparine; IV, Intravenous; NSAID, Non-Steroidal Anti-Inflammatory Drugs. Statistical significance was assessed using the Mann-Whitney U test or Fisher’s exact test for binary values. §: Measured via self-report; not validated. Bold values indicate statistical significance (p < 0.05).

### Cortisol = Group + Time + Time*Group + error

Then we are interested if the + Time*Group component is statistically significative when compared to the HC behavior, which represents our null hypothesis.

## Results

### Study population

**Figure 3:**
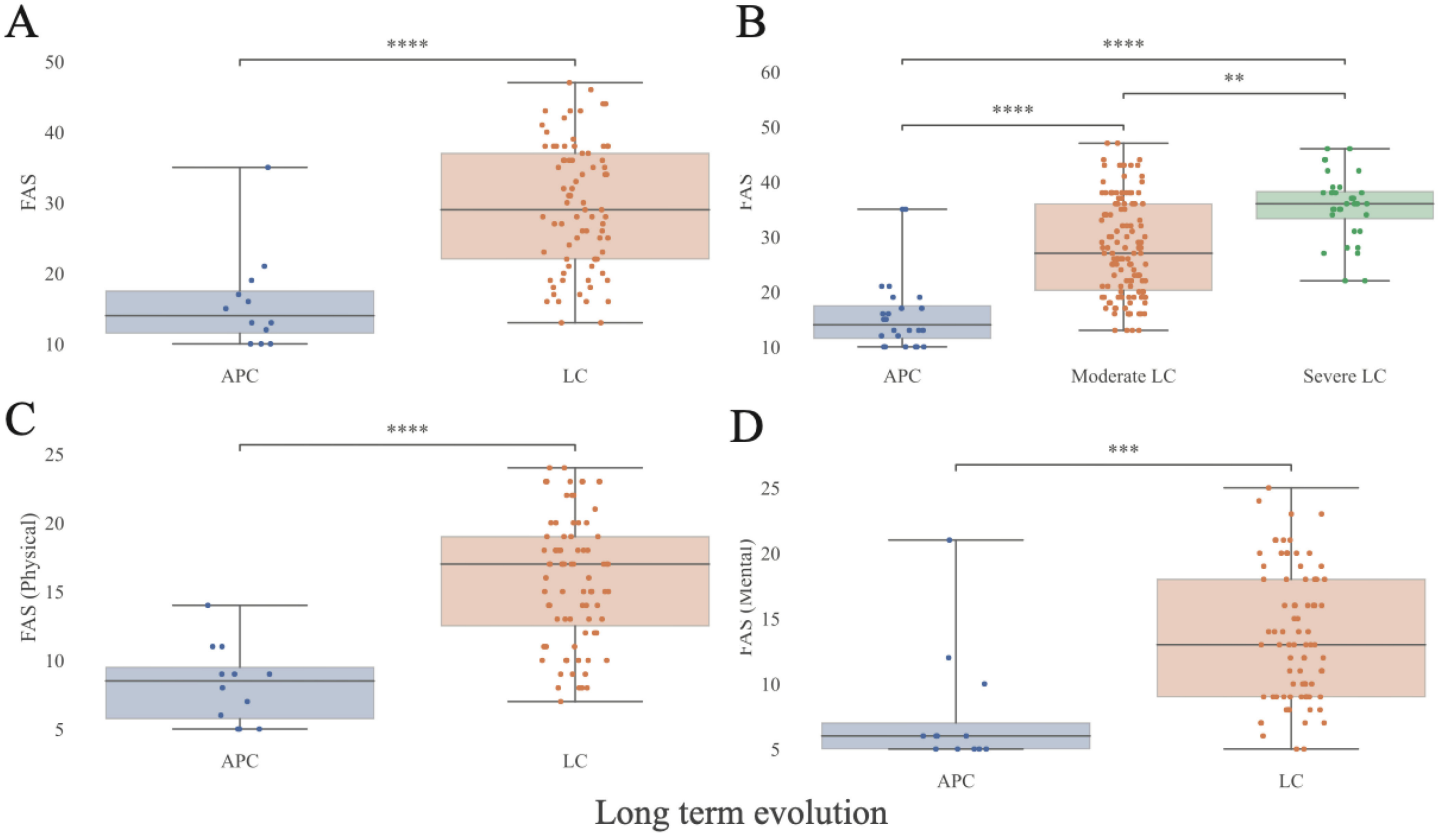
Fatigue Assessment Scale (FAS) score as an indicator of Long COVID symptoms. **(A)** FAS score distribution stratified by presence or absence of LC. **(B)** FAS score distribution further stratified by LC severity. **(C, D)** FAS score components (Physical, Mental) distributions stratified by presence or absence of LC. The FAS consists of 10 questions: 5 addressing physical fatigue and 5 addressing mental fatigue. A total score of ≥22 indicates the presence of fatigue, ≥35 defines extreme fatigue. APC, Asymptomatic Post-COVID, LC, Long-COVID. Statistical significance was assessed using the Mann-Whitney U test and values were corrected for FDR in a measure-specific manner (**: p<0.01; ***: p<0.001, ****: p<0.0001; APC N = 12, LC N = 79, Moderate LC N = 63, Severe LC N = 16; 5^th^, 25^th^, 50^th^, 75^th^, and 95^th^ quantiles marked). Extended data in **Supplementary Table S3**.

**Figure 4:**
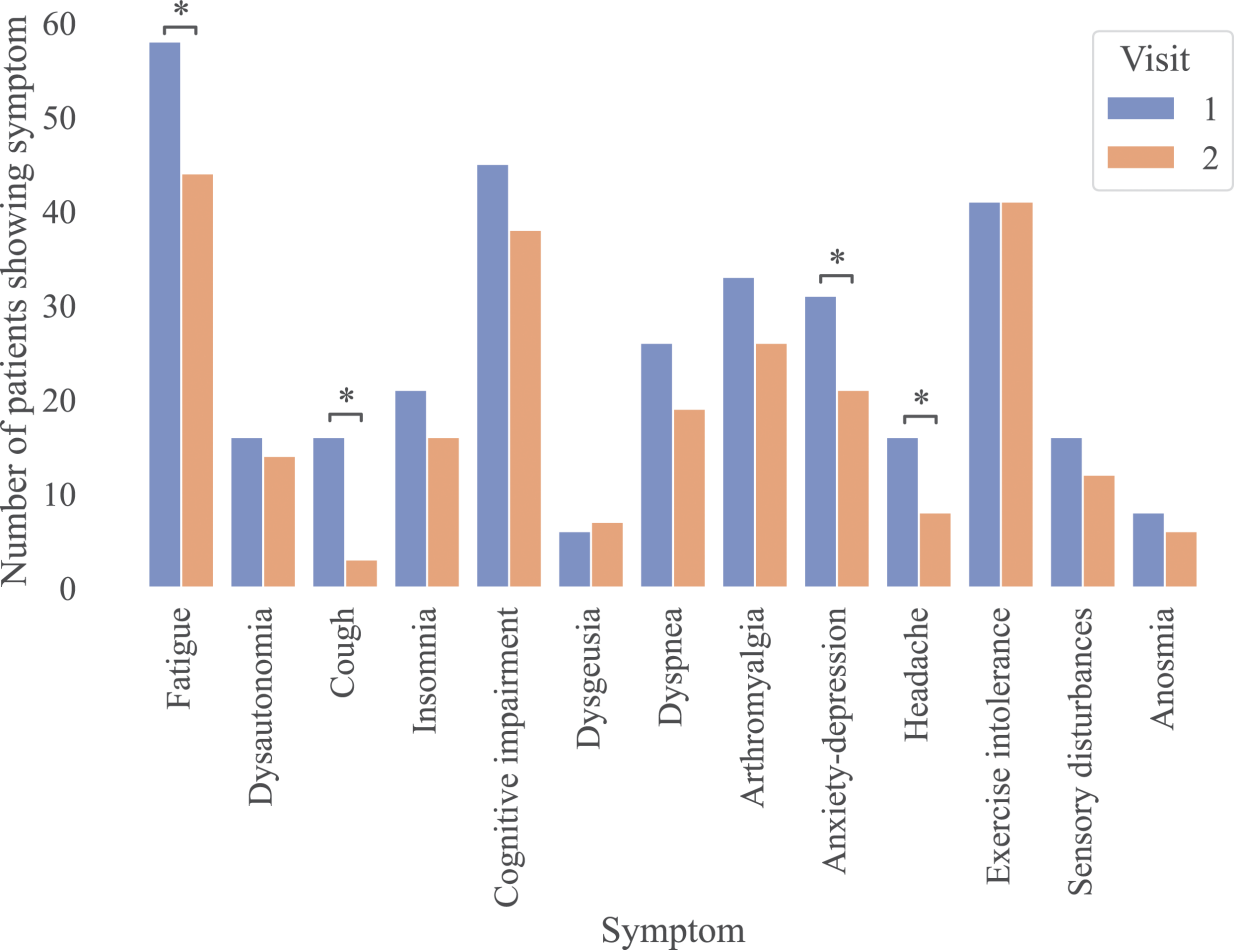
Prevalence of Long COVID symptoms at baseline and their progression three months after the initial diagnosis. N = 92 patients had a follow up second visit (Visit 2) after their initial assessment (baseline, Visit 1). Statistical significance was assessed using the Wilcoxon test and p-values were corrected for FDR across symptoms (*: p<0.05; **: p<0.01; ***: p<0.001, ****: p<0.0001).

**Table 1** presents the demographic and clinical characteristics of the 96 participants. The cohort included 60% female, all of White ethnicity, with a mean age of 58.1 ± 14.8 years. Among the 83 participants classified as LC (86%), 80% had moderate disease (1–4 SOI) and 20% had severe disease (≥5 SOI). Thirteen participants (14%) were classified as APC. A mild or asymptomatic acute SARS-CoV-2 infection managed at home was reported by 64% of LC patients, compared with 54% of APC participants. APC individuals were more often hospitalized and treated with antivirals or required oxygen supplementation (23% vs 9.6%; p=0.039). LC patients exhibited a higher proportion of ≥3 comorbidities (65% vs 38%, p=0.02), prior SARS-CoV-2 infection (44.6% vs 23%, p=0.03), and previous engagement in competitive sports (51.8% vs 30.1%, p=0.04). No significant differences were found in drug exposure between groups (**Table 2****).** Mean FAS scores were significantly higher in LC than in APC (29.3 vs 14.4, p<0.001), including both mental (13.5 vs 7.5, p<0.001) and physical domains (15.7 vs 8, p<0.001). At BL, 66 patients (24 males, 42 females) reported impairment in daily activities due to LC. FAS scores were strongly associated with LC diagnosis and severity (p<0.0001), and with reduced quality of life (p<0.001) (**Figures 3A-D**). Symptom prevalence at BL and V2 is shown in **Figure 4**.

**Table 2:** Baseline medication exposure among study participants.

Medications*	LC (N = 83)	APC (N = 13)	P-value
Anxiolytics/Hypnotics	8 (9.6%)	1 (7.7%)	1
Antibiotics	4 (4.8%)	0	0.69
Antihistamines	6 (7.2%)	0	0.69
Levothyroxine	17 (20.5%)	2 (15.4%)	1
Proton pump inhibitors	14 (16.9%)	2 (15.4%)	1
Antiarrhythmics	15 (18.1%)	0	0.21
Anticoagulants	10 (12%)	0	0.34
Antiplatelet agents	10 (12%)	1 (7.7%)	1
Antidepressants	11 (13.3%)	0	0.35
Lipid-lowering agents	13 (15.7%)	3 (23.1%)	0.45
Antihypertensives	24 (28.9%)	4 (30.8%)	1
Anticonvulsants	1 (1.2%)	0	1
Antiresorptive agents	3 (3.6%)	0	1
Antivirals	3 (3.6%)	0	1
Antidiabetic agents	7 (8.4%)	0	0.58
NSAIDs	6 (7.2%)	0	0.69
Bronchodilators	5 (6%)	1 (7.7%)	1
Cholecalciferol	26 (31.3%)	1 (7.7%)	0.1
Hypouricemic agents	1 (1.2%)	1 (7.7%)	0.25
5-alpha-reductase inhibitors	4 (4.8%)	0	1

*n (%). APC, Asymptomatic Post-COVID; LC, Long-COVID; NSAIDs, Non-Steroidal Anti-Inflammatory Drugs. Statistical significance was assessed using the Fisher’s exact test for binary values.

### Salivary circadian rhythm

The following analyses should be interpreted as exploratory due to the small HC sample. SC levels differed significantly among study groups (**Figure 2A**). HC exhibited significantly higher median 8:00 AM SC levels (18.54 nmol/L [IQR 3.3]) compared with both APC (13.16 nmol/L [IQR 6.97]; p=0.014) and LC participants (11.72 nmol/L [IQR 5.41]; p = 0.004). At 3:00 PM, median SC values were 2.92 nmol/L [IQR 2.44] in HC, 4.47 nmol/L [IQR 0.93] in APC, and 4.31 nmol/L [IQR 2.47] in LC (both p=1 vs HC). At 11:00 PM, corresponding values were 1.56 nmol/L [IQR 1.24] vs 2.25 nmol/L [IQR 0.8] in APC (p=0.18), and 2.27 nmol/L [IQR 1.65] in LC (p=0.05). When stratified by LC severity (**Figure 2B**), participants with severe LC exhibited higher median 11:00 PM SC levels (3.15 nmol/L [IQR 2.6]) compared with moderate LC (2.06 nmol/L [IQR 1.3]; p=0.67), APC (2.25 nmol/L; p=1) and HC (1.56 nmol/L; p=0.22). Morning SC levels were significantly lower in LC compared with HC, but no difference was found between moderate and severe LC subgroups (**Figure 2B**). Our linear mixed model approach revealed that the daily decrease in cortisol is more pronounced in HC than any other population (APC, LC, Severe LC, Moderate LC, **Supplementary Figure S1**). This result underlines the possibility of a reduction in amplitude of the salivary cortisol oscillations after exposure to SARS-CoV-2.

### HPA axis and other hormonal findings

**Figure 5:**
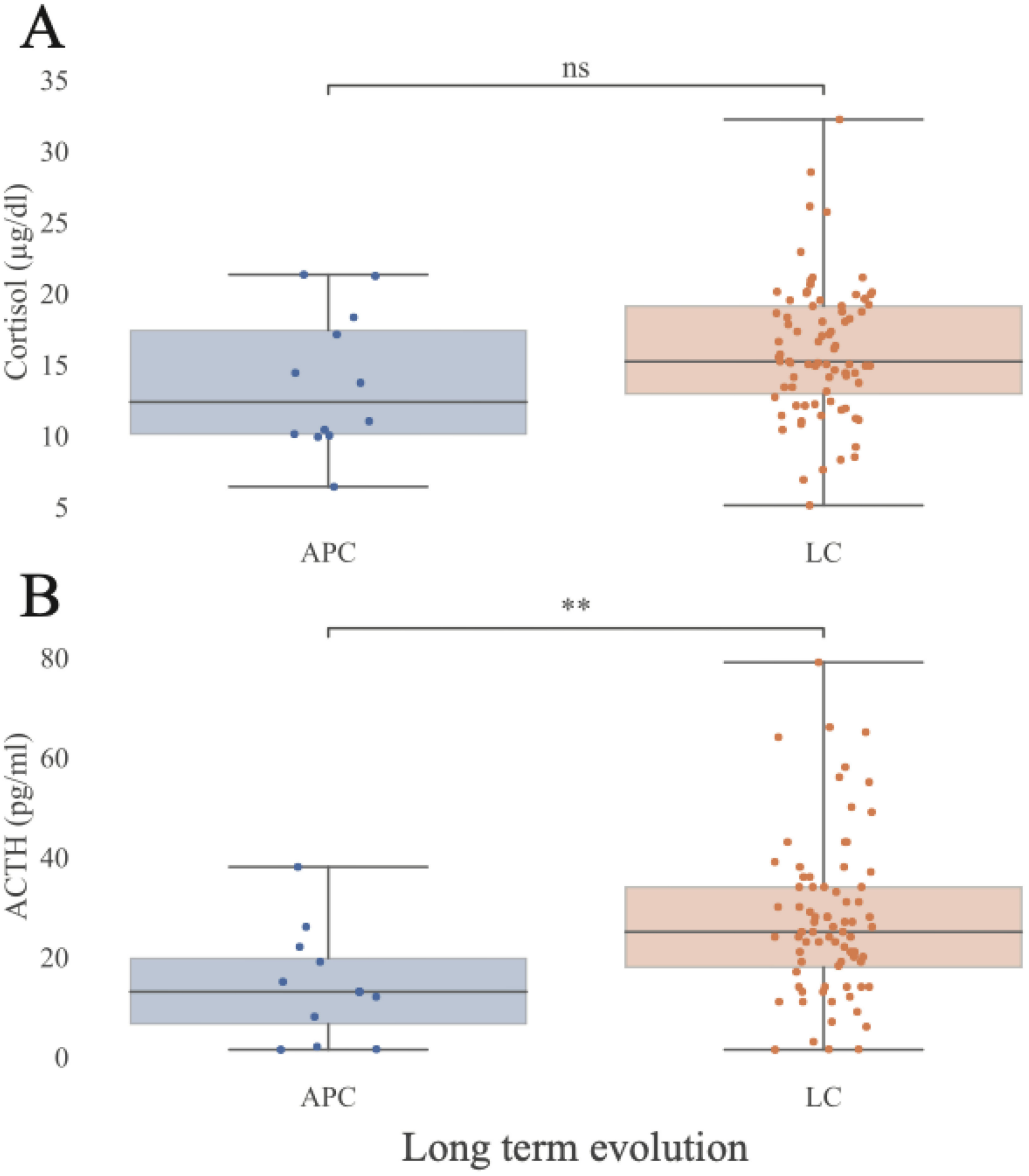
Pituitary and adrenal hormones distributions as a function of Long COVID diagnosis. Measurements stratified by presence or absence of LC. **(A)** Morning blood cortisol. **(B)** Morning blood ACTH. APC, Asymptomatic Post-COVID, LC, Long-COVID. ACTH, adrenocorticotropic hormone (pg/ml). Cortisol (μg/dl). Statistical significance was assessed using the Mann-Whitney U test and values were corrected for FDR in a measure-specific manner (**: p<0.01; Cortisol: APC N = 12, LC N = 79; 5^th^, 25^th^, 50^th^, 75^th^, and 95^th^ quantiles marked). Extended data in **Supplementary Table S5**.

**Figure 6:**
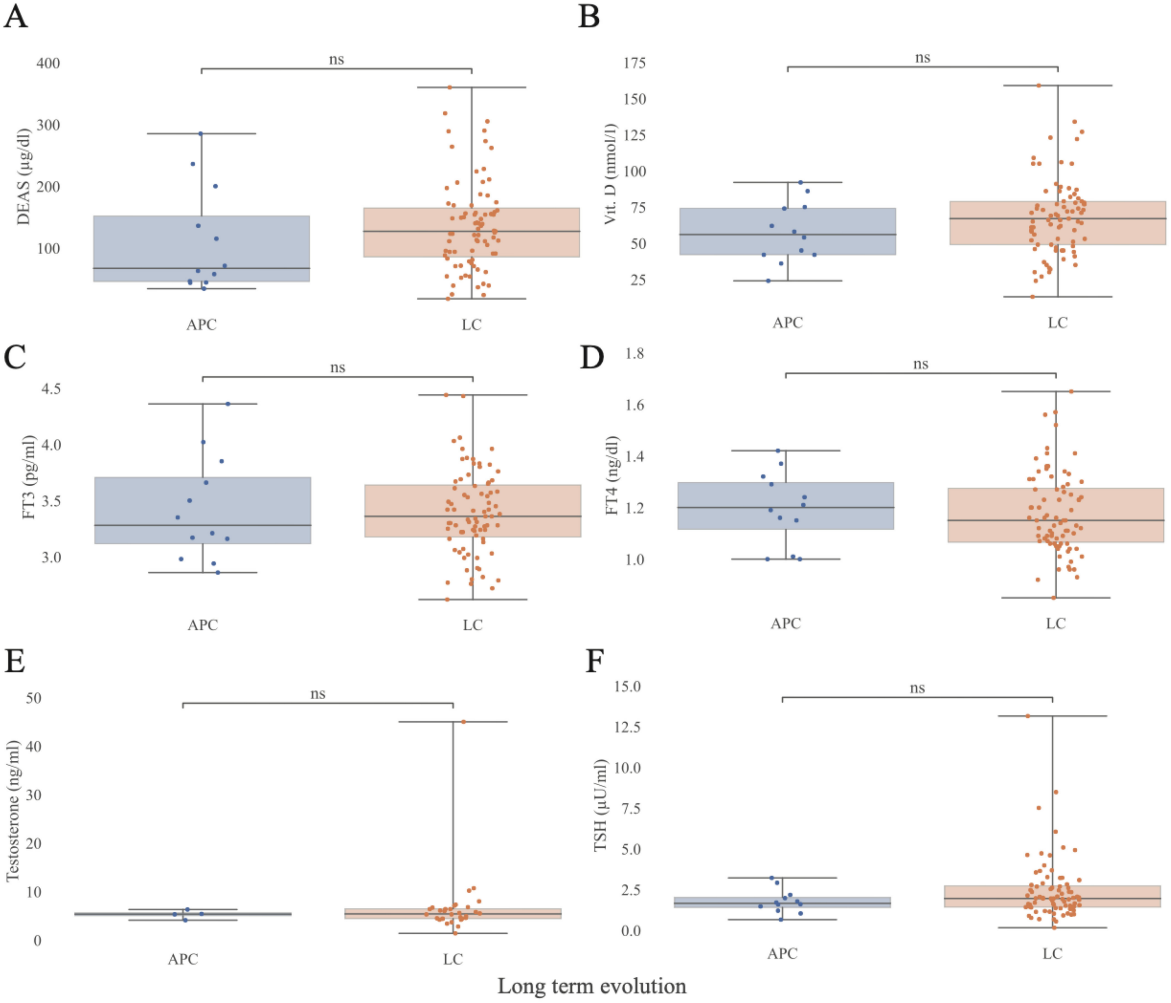
Androgens hormones, thyroids hormones and vitamin D distributions as a function of Long COVID. **(A–F)** Stratification by presence or absence of LC. APC, Asymptomatic Post-COVID; LC, Long-COVID; DHEAS, dehydroepiandrosterone sulfate (µg/dl); TSH, thyroid-stimulating hormone(µU/ml); fT3, free-T3 (pg/ml). fT4 free-T4 (ng/dl). Testosterone (ng/ml) was tested only for male participants. Vit. D: Vitamin D (nmol/l). Statistical significance was assessed using the Mann-Whitney U test and values were corrected for FDR in a measure-specific manner (APC N = 12, LC N = 79; Testosterone: APC N = 4, LC N = 34; 5^th^, 25^th^, 50^th^, 75^th^, and 95^th^ quantiles marked). Extended data in **Supplementary Table S6**.

Morning BC levels did not differ significantly between LC and APC participants (15.2 µg/dL [IQR 6.2] vs 12.3 µg/dL [IQR 7.4] p= 0.1). Median ACTH hormone concentrations were higher in LC compared with APC group (25 pg/mL [IQR 16.1] vs 13 pg/mL [IQR 13.2]; p = 0.003), while remaining within normal laboratory reference ranges (**Figures 5A, B****).** Thirty-two patients (26 with LC [21.6% of all LC patients] and 6 APC [46% of all APC patients]) met the inclusion criteria for the ACTH-test; five declined participation. Among the 27 individuals tested, one female LC patient showed a subnormal cortisol response (BC 0’ 9.6 µg/dL, BC 30’ 9.2 µg/dL, BC 60’ 14.5 µg/dL) and was diagnosed with adrenal insufficiency requiring replacement therapy. No statistically significant differences were found between APC and LC groups for testosterone (males only), DHEAS, vitamin D, TSH, fT3, or fT4 (**Figures 6A–F****).** Median (IQR) values for APC and LC, respectively, were as follows: testosterone 5.35 ng/ml (IQR 0.62) vs 5.4 ng/ml (IQR 2.07) p=0.7; DHEAS 67.05 µg/dL (IQR 106.3) vs 127 µg/dL (IQR 79.5)p=0.14; vitamin D 56 nmol/L (IQR 32.2) vs 67 nmol/L (IQR 30) p= 0.2; TSH 1.65 µU/ml (IQR 0.63) vs 1.95 (IQR 1.32) p=0.29; fT3 3.28 pg/ml (IQR 0.6) vs 3.36 pg/ml (IQR 0.47) p= 0.8; and fT4 1.2 ng/dl (IQR 0.18) vs 1.15 ng/dl (IQR 0.21) p= 0.51.

### Inflammatory and coagulative findings

**Figure 7:**
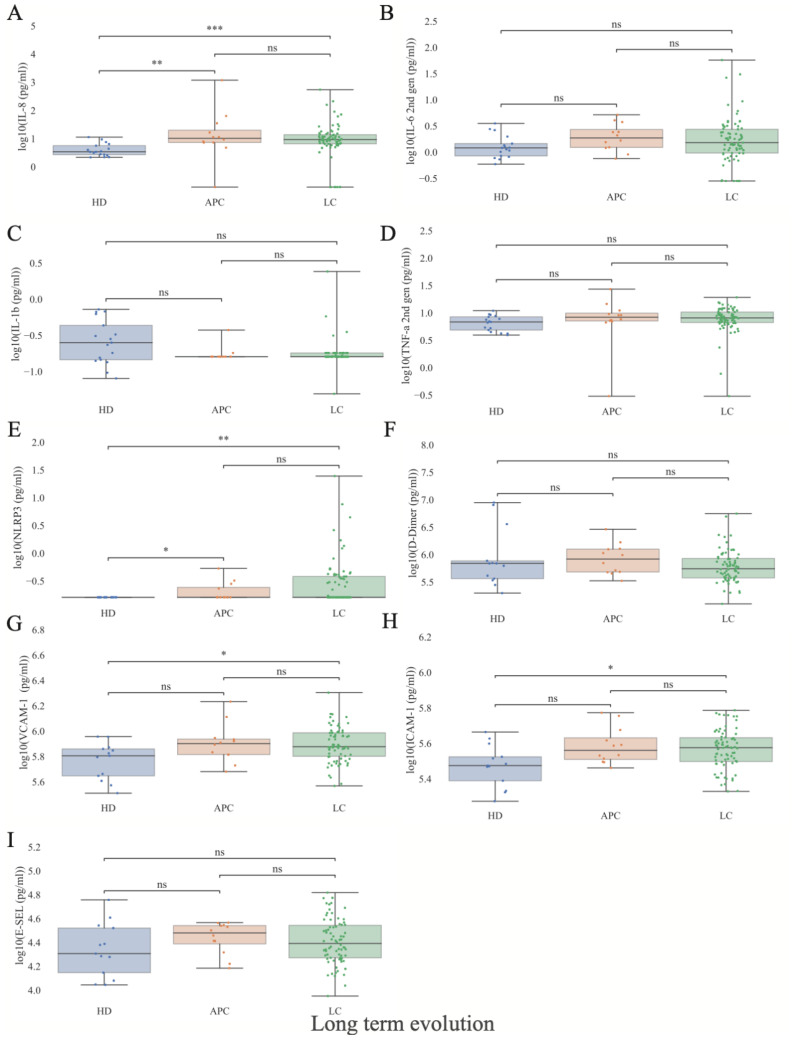
Inflammatory and coagulative markers distributions as a function of Long COVID. **(A–I)** Patient stratification by presence or absence of LC. Y-Axis in logarithmic scale. APC, Asymptomatic Post-COVID, LC, Long-COVID, HD, Healthy donors. IL-6, Interleukin-6 (pg/ml); IL-1β, Interleukin-1β (pg/ml); IL-8, Interleukin-8 (pg/ml). D-Dimer (pg/ml); ICAM-1: intercellular adhesion molecule-1 (pg/ml). VCAM-1: vascular cell adhesion molecule 1 (pg/ml). E-SEL: E-Selectine (pg/ml). NLRP3: NOD-like receptor family pyrin domain-containing 3 (pg/ml). TNF-α: tumor necrosis factor-alpha (pg/ml). Statistical significance was assessed using the Mann-Whitney U test and values were corrected for FDR in a measure-specific manner (*: p<0.05; **: p<0.01; ***: p<0.001, APC N = 12, LC N = 77; HD N = 18; 5^th^, 25^th^, 50^th^, 75^th^, and 95^th^ quantiles marked). Extended data in **Supplementary Table S7**.

(**Figures 7A–I**) presents the distribution of inflammatory and coagulation biomarkers among APC, LC, and HC participants. Both APC and LC subjects showed higher levels of inflammatory and endothelial activation markers compared with HC. Specifically, APC exhibited elevated median IL-8 levels (10.22 pg/ml [IQR 13.8] vs. 3.37 pg/ml [IQR 2.9] in HC; p=0.006) and NLRP3 levels (0.16 pg/ml [IQR 0] vs. 0.16 pg/ml [IQR 0.08] in HC; p=0.04), while LC participants showed similarly increased IL-8 (9.20 pg/ml [IQR 7.12] vs. 3.37 pg/ml [IQR 2.9] in HC; p=0.0001) and NLRP3 concentrations (0.16 pg/ml [IQR 0] vs. 0.16 pg/ml [IQR 0.22] in HC; p=0.003). No significant differences were observed for IL-1β, IL-6, or TNF-α across groups. Regarding coagulation and endothelial markers, LC participants exhibited higher median ICAM-1 levels (375896 pg/ml [IQR 114273] vs. 297954 pg/ml [IQR 89223] in HC; p=0.03) and VCAM-1levels (751944.5 pg/ml [IQR 337504] vs. 637439 [IQR 279772] in HC; p=0.01). APC subjects also showed elevated median ICAM-1 concentrations compared with HC (363694 pg/ml [IQR 1054133] vs. 297954 pg/ml [IQR 89223] in HC; p=0.09). No significant differences were observed for D-dimer or E-selectin across the three groups. No statistically significant differences in inflammatory or coagulation markers were detected between APC and LC participants.

### Follow-up visit (V2)

During the study, 4 patients were lost to follow-up, and 92 completed the V2 visit (3 months after BL). Mean total FAS scores significantly improved from BL to V2 (28 vs 24.5; p < 0.001), driven by improvement in the mental component (12.5 vs 10; p = 0.003), while physical FAS remained stable (15 vs 14; p = 0.7). At V2, 76 patients (82%) continued to meet the diagnostic criteria for LC (compared with 86% at BL). Among this, 18% were classified as severe and 81.5% as moderate.

## Discussion

Our findings indicate HPA axis dysregulation in individuals with LC, particularly among those with more severe symptomatology. Compared with APC and HC, LC participants exhibited a flattened circadian SC rhythm characterized by reduced morning and elevated evening/nighttime values, with more pronounced alterations in those with severe LC. This pattern may reflect impaired cortisol rhythmicity, consistent with adrenal strain and altered neuroendocrine feedback mechanisms.

Previous studies on cortisol in LC have yielded inconsistent results, likely reflecting methodological heterogeneity. A US cross-sectional study identified BC as the main predictor of LC, with an area under the curve (AUC) of 0.96, suggesting a central role for cortisol signaling in LC pathogenesis (Klein et al., 2023). Conversely, a German study reported no differences in BC levels among groups, although their broad sampling window (8–11 AM) and lack of wake-time standardization may have masked physiological peaks (Fleischer et al., 2024). A Thai cohort study reported hypocortisolism in 27% of patients three months after acute COVID-19, most of whom exhibited subnormal cortisol responses despite normal ACTH concentrations, findings consistent with secondary adrenal insufficiency. One case of primary adrenal failure, associated with elevated ACTH, was also identified. Among those with hypocortisolism, 45% remained symptomatic and required long-term glucocorticoid replacement therapy (Porntharukchareon et al., 2024). In our cohort, assessed at a mean of 155.5 days post-infection, one LC patient demonstrated an abnormal ACTH stimulation test despite normal basal ACTH levels, leading to a diagnosis of adrenal insufficiency likely involving both pituitary and adrenal dysfunction.

Our findings provide a more dynamic picture of cortisol secretion, capturing the circadian rhythm rather than a single time-point snapshot. Similarly, a preliminary report presented at CROI 2025 (abstract only, not peer-reviewed) on SC in LC, described a trend toward elevated ACTH levels (p = 0.06) and lower morning SC (p = 0.11) in LC patients comparing to APC, although the study lacked a healthy control group and stratification by LC severity (Annukka Antar et al., 2025).

This finding is consistent with our observation of increased ACTH concentrations despite normal plasma cortisol, suggesting compensatory pituitary activation and possible adrenal resistance.

Post-mortem studies have further substantiated these findings, detecting viral RNA and nucleocapsid protein in cerebral tissues for up to four months after SARS-CoV-2 infection (Leow et al., 2005), with replication competence confirmed in the hypothalamus (Stein et al., 2022). These observations support the hypothesis of viral persistence and long-term neuroendocrine involvement.

Notably, SARS-CoV mainly targets the hypothalamus and pituitary, while SARS-CoV-2 appears to preferentially affect the adrenal glands, which express ACE2 and TMPRSS2 receptors (Stein et al., 2022). Accordingly, functional (Stein et al., 2022)F-FDG PET/CT imaging confirmed reduced adrenal activity during both the acute and post-acute phase of SARS-CoV-2 infection (Lauri et al., 2023). Moreover, elevated BC in acute COVID, may reflect stress-induced cortisol release or direct adrenal lysis (Tan et al., 2020). Moreover, our results suggest that assessing SC profiles may serve as a non-invasive tool to identify patients with ongoing neuroendocrine dysfunction. Salivary cortisol, in fact, shows greater sensitivity than serum cortisol in detecting subtle alterations in HPA axis dynamics, as it reflects the unbound, biologically active fraction, is unaffected by corticosteroid-binding globulin variability, and allows for repeated, home-based sampling without venipuncture-related stress, thereby preserving circadian patterns (Tornhage, 2009; El-Farhan et al., 2024).

Taken together, these observations suggest that SARS-CoV-2 may interfere with stress-axis regulation at multiple levels, resulting in a mixed pattern of functional hypocortisolism. This complex phenomenon can be further understood by examining the intricate interplay among the HPA-axis, the autonomic nervous system (ANS), and immune responses (Pellissier et al., 2014). Dysautonomia is a frequent feature in LC patients, manifesting as orthostatic intolerance, tachycardia, or postural orthostatic tachycardia syndrome (POTS) (DePace and Colombo, 2022). A reduced vagal tone, commonly associated with dysautonomia, may disrupt the feedback between the ANS and endocrine stress systems, thereby contributing to flattened cortisol rhythms seen in LC (Pellissier et al., 2014; Livieratos et al., 2025). Experimental data further support this, demonstrating a bidirectional relationship between vagal activity and cortisol dynamics. Specifically, lower heart rate variability (HRV) has been associated with higher evening cortisol levels and loss of circadian amplitude (Pellissier et al., 2014). While our study did not directly assess HRV, previous investigations have reported significant reductions in LC (da Silva et al., 2023), supporting the hypothesis of combined autonomic–endocrine dysregulation in the condition (Acanfora et al., 2022; Camici et al., 2024). Beyond the ANS, inflammatory cytokines also play a crucial role in modulating HPA axis activity and circadian regulation. In both LC and APC individuals, we observed elevated levels of IL-8 and NLRP3 compared to healthy controls, indicating persistent immune activation even after clinical recovery from acute infection. This chronic exposure to cytokines can impair cortisol synthesis and disrupt melatonin production, contributing significantly to circadian misalignment (Loh and Reiter, 2022).

### Additional mechanisms that may contribute to HPA axis dysregulation in LC

Large-scale studies have underscored the adrenal glands and GR activity as major determinants of systemic circadian alignment (Talamanca et al., 2023), and women show stronger circadian gene expression, which may contribute to their greater susceptibility to LC (Talamanca et al., 2023). SARS-CoV-2 can induce epigenetic changes affecting both GR pathways and circadian genes, potentially prolonging HPA axis dysfunction in vulnerable individuals (Li et al., 2021). Similar GR desensitization has been observed in athletes (Yeager et al., 2011; Lu et al., 2017), among whom persistent symptoms after COVID-19 have been described (Diebold et al., 2025). In addition, SARS-CoV-2 has been shown to impair mitochondrial oxidative phosphorylation and increase ROS production, changes that can reduce cortisol synthesis and affect its intracellular signaling (Midzak and Papadopoulos, 2016; Zhang et al., 2020; Miller et al., 2021; Nunn et al., 2022; Mantle et al., 2024). Furthermore, the GR itself is sensitive to the cellular redox environment (Okamoto et al., 1999; Vandevyver et al., 2014). These redox-dependent mechanisms have been documented in steroid resistance syndromes and could similarly contribute to blunted cortisol action in LC. Beyond direct viral effects, molecular mimicry between ACTH and SARS-CoV-2 antigens has been proposed as an additional mechanism (Wheatland, 2004), whereby cross-reactive antibodies could attenuate ACTH activity, similar to patterns described in other coronavirus infections (Leow et al., 2005). Together, these alterations may help explain blunted cortisol action in LC and warrant targeted investigation.

### Study limitations

This study has several limitations. The very small number of healthy controls inherently reduces statistical robustness of our findings as such, the study should be considered exploratory. Home-based salivary cortisol collection may introduce uncontrolled variance due to behavioral factors. Because wake-up time and sleep patterns were not recorded, the observed reduction in morning SC may partly reflect inter-individual variability rather than intrinsic HPA axis dysfunction. Moreover, we did not control for psychological distress or physical activity, both of which influence cortisol secretion. The observed cortisol differences might also reflect secondary consequences of chronic illness rather than primary HPA axis dysregulation. Additionally, the single-center design may limit the generalizability of the data. Longitudinal studies with comprehensive covariate adjustment are needed to disentangle these relationships. The study also relied on an earlier definition of Long COVID (>4 weeks post-infection), which may limit comparability with research using the current WHO definition (within 3 months). Some measures, including smoking status and prior competitive sports participation, were qualitative and not assessed through validated questionnaires, which warrants cautious interpretation. Additionally, antidepressant use may theoretically influence HPA axis activity, but such effects are usually transient and normalize with chronic treatment. As treatment duration was not recorded, residual confounding cannot be fully excluded. Furthermore, several mechanistic aspects discussed, such as glucocorticoid receptor sensitivity, mitochondrial function, and circadian gene regulation, were not directly assessed. Finally, the observational design precludes causal inference, and the associations observed should be interpreted with caution. Despite these limitations, this is, to our knowledge, the first study to comprehensively characterize circadian SC profiles in LC. Future research should aim to confirm these findings in longitudinal cohorts integrating hormonal, immune, and neurocognitive assessments to better delineate post-COVID pathophysiology. Studies evaluating whether normalization of cortisol rhythmicity parallels clinical improvement will be crucial to understanding the reversibility and clinical significance of HPA axis dysfunction in LC. Moreover, to fully understand the abnormalities occurring in the HPA axis during LC and quantify their impact on symptomatology, it is necessary to further investigate genetic and epigenetic markers of GR sensitivity, as well as the mitochondrial function of endocrine cells within the HPA axis in these patients.

## Conclusions

In summary, our study demonstrates that patients with LC exhibit a flattened circadian SC rhythm, characterized by reduced morning and elevated evening levels, along with higher ACTH concentrations compared with asymptomatic post-COVID and healthy controls. The disruption of cortisol rhythmicity was more pronounced in individuals with severe LC, suggesting a potential link between hormonal imbalance and symptom burden. This pattern may reflect a loss of normal circadian regulation of the stress axis, consistent with compensatory pituitary activation or impaired adrenal responsiveness. SC profiling emerges as a simple, non-invasive approach to assess HPA axis function in post-COVID conditions. Although this study cannot establish causality, the data support a model in which persistent neuroendocrine dysregulation contributes to the heterogeneity of LC. Longitudinal studies are needed to validate these associations, define reference ranges for post-viral HPA recovery, and clarify whether restoration of cortisol rhythmicity corresponds with clinical improvement.

## Data Availability

The original contributions presented in the study are included in the article/**Supplementary Material**. Further inquiries can be directed to the corresponding author.
