# Supplementary material for: Salivary cortisol in long COVID: a marker of broader stress system and circadian rhythm dysregulation
Source: Front Cell Infect Microbiol. 2026 Jan 6;15:1690698. doi: 10.3389/fcimb.2025.1690698 (PMC12816214; doi:10.3389/fcimb.2025.1690698)
Supplement: Supplementary file 1 [file DataSheet1.pdf]

```
                        Mixed Linear Model Regression Results
==========================================================================================
Model:                        MixedLM            Dependent Variable:            value
No. Observations:             255                Method:                        REML
No. Groups:                   90                 Scale:                         12.6861
Min. group size:              1                  Log-Likelihood:                -690.7997
Max. group size:              3                  Converged:                     Yes
Mean group size:              2.8
------------------------------------------------------------------------------------------
                                               Coef.  Std.Err.   z    P>|z|  [0.025 0.975]
------------------------------------------------------------------------------------------
Intercept                                      24.629    2.109 11.678 0.000  20.495 28.762
C(group, Treatment(reference='HC'))[T.APC]     -8.368    2.750 -3.043 0.002 -13.757 -2.978
C(group, Treatment(reference='HC'))[T.LC]      -7.824    2.216 -3.530 0.000 -12.168 -3.480
time                                           -1.098    0.127 -8.654 0.000  -1.346 -0.849
time:C(group, Treatment(reference='HC'))[T.APC]  0.452    0.165  2.732 0.006   0.128  0.776
time:C(group, Treatment(reference='HC'))[T.LC]   0.433    0.133  3.250 0.001   0.172  0.695
Group Var                                       0.432    0.264
==========================================================================================
```

A

```
                           Mixed Linear Model Regression Results
==============================================================================================
Model:                            MixedLM            Dependent Variable:            value
No. Observations:                 255                Method:                        REML
No. Groups:                       90                 Scale:                         12.6955
Min. group size:                  1                  Log-Likelihood:                -688.4135
Max. group size:                  3                  Converged:                     Yes
Mean group size:                  2.8
----------------------------------------------------------------------------------------------
                                                        Coef.  Std.Err.   z    P>|z|  [0.025 0.975]
----------------------------------------------------------------------------------------------
Intercept                                               24.629    2.101 11.722 0.000  20.511 28.747
C(group, Treatment(reference='HC'))[T.APC]              -8.368    2.740 -3.054 0.002 -13.737 -2.998
C(group, Treatment(reference='HC'))[T.Moderate LC]      -8.412    2.231 -3.771 0.000 -12.784 -4.040
C(group, Treatment(reference='HC'))[T.Severe LC]        -5.123    2.643 -1.938 0.053 -10.304  0.058
time                                                    -1.098    0.127 -8.651 0.000  -1.346 -0.849
time:C(group, Treatment(reference='HC'))[T.APC]          0.452    0.165  2.731 0.006   0.128  0.776
time:C(group, Treatment(reference='HC'))[T.Moderate LC]  0.454    0.135  3.367 0.001   0.189  0.718
time:C(group, Treatment(reference='HC'))[T.Severe LC]    0.339    0.160  2.119 0.034   0.025  0.652
Group Var                                                0.180    0.251
==============================================================================================
```

B

Figure S1: linear mixed model results. Screenshots directly from code summary in python. These reports are always compared to the HC which we assume to be our null hypothesis whose parameters are expressed in the `Intercept` and `time` rows. For these rows the shown p-value is just assumin a null hypothesis of the parameter being equal to 0. **(A) Results of the model when considering Moderate LC and LC sparated together as LC.** Adj. p-values: {Intercept<= 0.001; C(APC)=0.003,C(LC)<=0.001; time<=0.001; time:C(APC)=0.006; time:C(LC)=0.002} **(B) Results of the model when considering Moderate LC and LC sparated.** Adj. p-values: { Intercept<= 0.002; C(APC)=0.003; C(Moderate LC)<0.002; C(Moderate LC)=0.053; time <= 0.002; time:C(APC)=0.008, time:C(Moderate LC)=0.002; time:C(Moderate LC)=0.039}.

For this model we took as the null hypothesis that the daily slope of cortisol was the same among HC and various long covid types. We fitted a mixed linear model to understand if the slope of daily decrease was constant across all patients or differed for the long covid group. The 'Intercept' and 'time' rows are the result of the fit for the HC group; the other rows are comparing the fit of a specific group to HC. For example, if we are interested in the behaviour of the APC population we can look at line `C(group, Treatment(reference='HC'))[T.APC]` to understand the numerical and statistical differences in mean value across the day between the two populations and at line `time:C(group, Treatment(reference='HC'))[T.APC]` to assess the daily decrease and investigate if cortisol decreases at the same rate in the two populations. Both lines report the difference in value (by some called effect size) between the HC and APC for each parameter in column `Coeff.` as well as other parameters of the fit, in particualr the p.value of the two fits being the same in column `P>|z|`.

Exam	Tested pair	Adjusted p-value	Raw p-value	5, 25, 50, 75, 95 quantiles 1st	5, 25, 50, 75, 95 quantiles 2nd
FAS	APC - Moderate LC	9.30047E-05	3.10016E-05	[10. 11.5 14. 17.5 27.3]	[16. 20.5 27. 36. 43.]
FAS	Moderate LC - Severe LC	0.009369835	0.003123278	[16. 20.5 27. 36. 43.]	[25.75 33.25 36. 38.25 44.5]
FAS	APC - Severe LC	9.49733E-05	3.16578E-05	[10. 11.5 14. 17.5 27.3]	[25.75 33.25 36. 38.25 44.5]
FAS	APC - LC	9,0857E-06	9,0857E-06	[10. 11.5 14. 17.5 27.3]	[16. 22. 29. 37. 43.1]
FAS (Mental)	APC - LC	0,000115229	0,000115229	[5. 5. 6. 7. 16.05]	[7. 9. 13. 18. 21.2]
FAS (Physical)	APC - LC	4,05406E-06	4,05406E-06	[5. 5.75 8.5 9.5 12.35]	[8. 12.5 17. 19. 23.]

Supplementary Table S3. Numerical values of Adj. P-values and 5th, 25th, 50th, 75th, and 95th quantiles for variables, distributions, and comparisons shown in figure 3. APC: Asymptomatic Post-COVID, LC: Long-COVID.

Exam	Tested pair	Adj. pval	Raw pval	5, 25, 50, 75, 95 quantiles 1st	5, 25, 50, 75, 95 quantiles 2nd
SC 8:00	APC - Moderate LC	1	0.764177156	[5.09 8.265 13.165 15.2325 17.465]	[6.37 9.57 11.1 14.055 20.914]
SC 8:00	Moderate LC - Severe LC	0.733728995	0.122288166	[6.37 9.57 11.1 14.055 20.914]	[6.989 11.3225 14.205 17.5625 26.7785]
SC 8:00	Severe LC - Control	1	0.195562436	[6.989 11.3225 14.205 17.5625 26.7785]	[14.523 16.475 18.54 19.77 20.984]
SC 8:00	APC - Severe LC	1	0.448278862	[5.09 8.265 13.165 15.2325 17.465]	[6.989 11.3225 14.205 17.5625 26.7785]
SC 8:00	Moderate LC - Control	0.002362442	0.00039374	[6.37 9.57 11.1 14.055 20.914]	[14.523 16.475 18.54 19.77 20.984]
SC 8:00	APC - Control	0.027766351	0.004627725	[5.09 8.265 13.165 15.2325 17.465]	[14.523 16.475 18.54 19.77 20.984]
SC 15:00	APC - Moderate LC	1	0.465258345	[2.86 4.26 4.47 5.1925 7.5565]	[2.048 3.03 4.09 5.26 8.98]
SC 15:00	Moderate LC - Severe LC	1	0.612890974	[2.048 3.03 4.09 5.26 8.98]	[2.318 2.79 4.53 6.69 8.226]
SC 15:00	Severe LC - Control	1	0.427936939	[2.318 2.79 4.53 6.69 8.226]	[2.574 2.725 2.92 5.165 6.001]
SC 15:00	APC - Severe LC	1	0.925864602	[2.86 4.26 4.47 5.1925 7.5565]	[2.318 2.79 4.53 6.69 8.226]
SC 15:00	Moderate LC - Control	1	0.525708976	[2.048 3.03 4.09 5.26 8.98]	[2.574 2.725 2.92 5.165 6.001]
SC 15:00	APC - Control	1	0.36384204	[2.86 4.26 4.47 5.1925 7.5565]	[2.574 2.725 2.92 5.165 6.001]
SC 23:00	APC - Moderate LC	1	0.812855241	[1.5215 1.8725 2.255 2.67 2.762]	[1.4 1.535 2.06 2.835 3.835]
SC 23:00	Moderate LC - Severe LC	0.406449016	0.067741503	[1.4 1.535 2.06 2.835 3.835]	[1.4495 1.8 3.15 4.405 7.996]
SC 23:00	Severe LC - Control	0.133196929	0.022199488	[1.4495 1.8 3.15 4.405 7.996]	[0.5 0.5 1.56 1.74 2.597]
SC 23:00	APC - Severe LC	1	0.198262144	[1.5215 1.8725 2.255 2.67 2.762]	[1.4495 1.8 3.15 4.405 7.996]
SC 23:00	Moderate LC - Control	0.15519752	0.025866253	[1.4 1.535 2.06 2.835 3.835]	[0.5 0.5 1.56 1.74 2.597]
SC 23:00	APC - Control	0.377383474	0.062897246	[1.5215 1.8725 2.255 2.67 2.762]	[0.5 0.5 1.56 1.74 2.597]
SC 8:00	Control - APC	0.013883	0.004628	[14.523 16.475 18.54 19.77 20.984]	[5.09 8.265 13.165 15.2325 17.465]
SC 8:00	APC - LC	1	0.945635	[5.09 8.265 13.165 15.2325 17.465]	[6.25 9.645 11.72 15.025 21.593]
SC 8:00	Control - LC	0.004237	0.001412	[14.523 16.475 18.54 19.77 20.984]	[6.25 9.645 11.72 15.025 21.593]
SC 15:00	Control - APC	1	0.363842	[2.574 2.725 2.92 5.165 6.001]	[2.86 4.26 4.47 5.1925 7.5565]
SC 15:00	APC - LC	1	0.526836	[2.86 4.26 4.47 5.1925 7.5565]	[2.0285 3. 4.31 5.47 8.6055]
SC 15:00	Control - LC	1	0.478456	[2.574 2.725 2.92 5.165 6.001]	[2.0285 3. 4.31 5.47 8.6055]
SC 23:00	Control - APC	0.188692	0.062897	[0.5 0.5 1.56 1.74 2.597]	[1.5215 1.8725 2.255 2.67 2.762]
SC 23:00	APC - LC	1	0.927457	[1.5215 1.8725 2.255 2.67 2.762]	[1.4 1.555 2.27 3.125 4.768]
SC 23:00	Control - LC	0.055061	0.018354	[0.5 0.5 1.56 1.74 2.597]	[1.4 1.555 2.27 3.125 4.768]

Supplementary Table S4. Numerical values of Adj. P-values and 5th, 25th, 50th, 75th, and 95th quantiles for variables, distributions, and comparisons shown in figure 4. Adj: adjusted, Control: Healthy controls, APC: Asymptomatic Post-COVID, LC: Long-COVID, SC: Salivary Cortisol.

Exam	Tested pair	Adjusted p-value	Raw p-value	5, 25, 50, 75, 95 quantiles 1st	5, 25, 50, 75, 95 quantiles 2nd
Cortisol	APC - LC	0,105463211	0,105463211	[8.325 10.075 12.35 17.4 21.245]	[8.48 12.9 15.2 19.1 23.18]
ACTH	APC - LC	0,003573644	0,003573644	[1.4495 6.5 13. 19.75 31.4]	[5.25 17.9 25. 34. 59.5]

Supplementary Table S5. Numerical values of Adj. P-values and 5th, 25th, 50th, 75th, and 95th quantiles for variables, distributions, and comparisons shown in figure 5. Adj: adjusted, APC: Asymptomatic Post-COVID, LC: Long-COVID. ACTH: adrenocorticotropic hormone (pg/ml). Cortisol (μg/dl).

Exam	Tested pair	Adjusted p-value	Raw p-value	5, 25, 50, 75, 95 quantiles 1st	5, 25, 50, 75, 95 quantiles 2nd
DEAS	APC - LC	0,142562964	0,142562964	[39.425 45.675 67.05 152. 258.05]	[38.84 85.5 127. 165. 289.1]
Testosterone	APC - LC	0,705530249	0,705530249	[4.28 5. 5.35 5.625 6.165]	[3.13 4.4 5.4 6.475 10.425]
TSH	APC - LC	0,296486123	0,296486123	[0.859 1.395 1.65 2.02 3.045]	[0.752 1.41 1.95 2.735 5.177]
FT3	APC - LC	0,869546201	0,869546201	[2.904 3.115 3.28 3.7075 4.173]	[2.788 3.175 3.36 3.64 3.967]
FT4	APC - LC	0,514893116	0,514893116	[1. 1.115 1.2 1.2975 1.3925]	[0.96 1.065 1.15 1.275 1.439]
Vit. D	APC - LC	0,21364607	0,21364607	[30.6 42. 56. 74.25 88.7]	[30. 49. 67. 79. 122.1]

Supplementary Table S6. Full numerical values corresponding to Figure 6, reporting median and interquartile range (IQR) for each variable across APC and LC groups. Adj: adjusted, APC: Asymptomatic Post-COVID, LC: Long-COVID. DHEAS: dehydroepiandrosterone sulfate (µg/dl). TSH: thyroid-stimulating hormone(µU/ml). fT3: free-T3 (pg/ml). fT4 free-T4 (ng/dl). Testosterone (ng/ml) was tested only for male participants. Vit. D: Vitamin D (nmol/l).

Exam	Tested pair	Adj p-value	Raw p-value	5, 25, 50, 75, 95 quantiles 1st	5, 25, 50, 75, 95 quantiles 2nd
IL-1b	HD - APC	0,895105	0.298368325	[0.0928 0.145 0.25 0.433 0.6842]	[0.16 0.16 0.16 0.16 0.26685]
IL-1b	APC - LC	1	0.398179992	[0.16 0.16 0.16 0.16 0.26685]	[0.16 0.16 0.16 0.18 0.2066]
IL-1b	HD - LC	0,254652	0.084884098	[0.0928 0.145 0.25 0.433 0.6842]	[0.16 0.16 0.16 0.18 0.2066]
IL-6	HD - APC	0,188648	0.062882648	[0.6966 0.842 1.2 1.45 2.8984]	[0.83115 1.2225 1.87 2.75 4.535]
IL-6	APC - LC	1	0.400337764	[0.83115 1.2225 1.87 2.75 4.535]	[0.28 0.956 1.51 2.72 8.228]
IL-6	HD - LC	0,413799	0.137932923	[0.6966 0.842 1.2 1.45 2.8984]	[0.28 0.956 1.51 2.72 8.228]
IL-8	HD - APC	0,006735	0.002245001	[2.182 2.675 3.371 5.59 9.72]	[2.687 7.2175 10.22 21. 562.5]
IL-8	APC - LC	1	0.576304672	[2.687 7.2175 10.22 21. 562.5]	[0.19 6.48 9.2 13.7 74.92]
IL-8	HD - LC	0,000155	5.16401E-05	[2.182 2.675 3.371 5.59 9.72]	[0.19 6.48 9.2 13.7 74.92]
TNF-α	HD - APC	0,218741	0.072913758	[4.1188 4.829 6.79 8.42 9.6936]	[3.7925 7.0775 8.31 9.8675 20.125]
TNF-α	APC - LC	1	0.723045608	[3.7925 7.0775 8.31 9.8675 20.125]	[4.396 6.63 8.06 10.3 14.02]
TNF-α	HD - LC	0,062083	0.020694472	[4.1188 4.829 6.79 8.42 9.6936]	[4.396 6.63 8.06 10.3 14.02]
NLRP3	HD - APC	0,040117	0.013372347	[0.16 0.16 0.16 0.16 0.16]	[0.16 0.16 0.16 0.2425 0.4145]
NLRP3	APC - LC	1	0.340464496	[0.16 0.16 0.16 0.2425 0.4145]	[0.16 0.16 0.16 0.38 1.88]
NLRP3	HD - LC	0,003759	0.001252947	[0.16 0.16 0.16 0.16 0.16]	[0.16 0.16 0.16 0.38 1.88]
D-Dimer	HD - APC	1	0.531631442	[245867.8 362545. 687919. 766435. 8330657.2]	[397030.1 479702.25 836435.5 1254027.75 2214075.95]
D-Dimer	APC - LC	0,319605	0.106534881	[397030.1 479702.25 836435.5 1254027.75 2214075.95]	[213785. 374067.75 549896. 846025.25 2138137.45]
D-Dimer	HD - LC	1	0.672544086	[245867.8 362545. 687919. 766435. 8330657.2]	[213785. 374067.75 549896. 846025.25 2138137.45]
E-SEL	HD - APC	0,49631	0.165436534	[11105.8 13987. 20211. 33105. 47115.8]	[15992.95 24492.5 30169.5 34748.5 36632.7]
E-SEL	APC - LC	1	0.496988023	[15992.95 24492.5 30169.5 34748.5 36632.7]	[13359.7 18630.5 24591.5 34903.75 53467.8]
E-SEL	HD - LC	0,876101	0.292033577	[11105.8 13987. 20211. 33105. 47115.8]	[13359.7 18630.5 24591.5 34903.75 53467.8]
ICAM-1	HD - APC	0,095018	0.031672727	[201446.6 244751. 297954. 333974. 438182.4]	[301262.2 322674. 363694. 428087.75 578943.8]

ICAM-1	APC - LC	1	0.807982488	[301262.2 322674. 363694. 428087.75 578943.8]	[241884.9 314030.75 375896.5 428303.25 573654.]
ICAM-1	HD - LC	0,031965	0.010655055	[201446.6 244751. 297954. 333974. 438182.4]	[241884.9 314030.75 375896.5 428303.25 573654.]
VCAM-1	HD - APC	0,181738	0.060579437	[353012.6 442702. 637439. 722474. 902254.4]	[509408.75 651543.5 795190.5 863740.25 1479008.]
VCAM-1	APC - LC	1	0.876199981	[509408.75 651543.5 795190.5 863740.25 1479008.]	[491306.75 631480. 751944.5 968984.5 1287584.7]
VCAM-1	HD - LC	0,013056	0.004351897	[353012.6 442702. 637439. 722474. 902254.4]	[491306.75 631480. 751944.5 968984.5 1287584.7]

Supplementary Table S7. Numerical values of Adj. P-values and 5th, 25th, 50th, 75th, and 95th quantiles for variables, distributions, and comparisons shown in figure 7. HC: Healthy controls, APC: Asymptomatic Post-COVID, LC: Long-COVID. IL-6: Interleukin-6 (pg/ml); IL-1β: Interleukin-1β (pg/ml); IL-8: Interleukin-8 (pg/ml). D-Dimer (pg/ml); ICAM-1: intercellular adhesion molecule-1 (pg/ml). VCAM-1: vascular cell adhesion molecule 1 (pg/ml). E-SEL: E-Selectine (pg/ml). NLRP3: NOD-like receptor family pyrin domain-containing 3 (pg/ml). TNF-α: tumor necrosis factor-alpha (pg/ml).
